# Aberrant E-cadherin staining patterns in invasive mammary carcinoma

**DOI:** 10.1186/1477-7819-3-73

**Published:** 2005-11-14

**Authors:** Malini Harigopal, Sandra J Shin, Melissa P Murray, Satish K Tickoo, Edi Brogi, Paul Peter Rosen

**Affiliations:** 1 Department of Pathology and Laboratory Medicine, New York Presbyterian Hospital Weill Cornell Medical Center, New York, NY, USA; 2 Department of Pathology, Memorial Sloan-Kettering Cancer Center, New York, NY USA

## Abstract

**Background:**

E-cadherin, a cell surface protein involved in cell adhesion, is present in normal breast epithelium, benign breast lesions, and in breast carcinoma. Alterations in the gene CDH1 on chromosome 16q22 are associated with changes in E-cadherin protein expression and function. Inactivation of E-cadherin in lobular carcinomas and certain diffuse gastric carcinomas may play a role in the dispersed, discohesive "single cell" growth patterns seen in these tumors. The molecular "signature" of mammary lobular carcinomas is the loss of E-cadherin protein expression as evidenced by immunohistochemistry, whereas ductal carcinomas are typically E-cadherin positive.

**Patients and methods:**

We report on E-cadherin immunostaining patterns in five cases of invasive mammary carcinoma

**Results:**

These were five exceptional instances in which the E-cadherin immunophenotype did not correspond to the apparent histologic classification of the lesion. These cases which are exceedingly rare in our experience are the subject of this report.

**Conclusion:**

Findings such as those illustrated in this study occur in virtually all biologic phenomena and they do not invalidate the very high degree of correlation between the expression of E-cadherin and the classification of breast carcinomas as ductal or lobular type on the basis of conventional histologic criteria.

## Background

The utility of the E-cadherin immunohistochemical stain to distinguish between lobular and ductal carcinomas that are difficult to classify by morphologic features alone has been well-documented in recent years [1-8]. However, greater experience in the staining patterns of lobular and ductal lesions with the E-cadherin stain has led to the discovery of rare instances of unexpected E-cadherin staining [1,6,7,9]. In this study, we report 5 cases of invasive mammary carcinoma with a striking discordance between the structural phenotype of the lesion in hematoxylin and eosin sections and immunohistochemical staining pattern for E-cadherin, a phenomenon we have termed as "aberrant".

## Patients and methods

Four cases were obtained from the Pathology department of the New York Presbyterian Hospital-Weill Medical College of Cornell University and a fifth case from Memorial Sloan Kettering Cancer Center, between Jan 1999 and July 2004. All specimens were available as formalin-fixed paraffin embedded tissue blocks and hematoxylin and eosin stained slides.

For E-cadherin immunostaining, four micron-thick sections were prepared from paraffin blocks containing lesional tissue. The slides were subsequently deparaffinized in three 5-minute changes of xylene and rehydrated through graded alcohols to distilled water. Heat induced epitope retrieval was performed on paraffin sections by pretreatment in a pressure cooker using 10 mM citrate buffer pH 6.0 for two minutes. Immunohistochemical staining was performed on paraffin sections using a TechMate 500 TM automated immunostainer (Ventana Medical Systems, Inc., Tuscon, AX) according to a modified MIP protocol (Ventana Medical System, Inc.) using the ChemMate ABC peroxidase secondary detection system (Ventana Medical Systems, Inc). A monoclonal antibody to E-cadherin, clone HECD-1 (Zymed Laboratories, Inc., San Francisco, CA) was used in 1:400 dilution. The peroxidase reaction was developed using DAB chromogen provided in the kit. Sections were counterstained with hematoxylin. The hematoxylin and eosin and immunohistochemical stained slides were subsequently reviewed and histopathologic features of lesional areas recorded.

## Results

A summary of the morphologic and immunohistochemical features of the following five cases is presented in Table 1.

**Table 1:** Histologic classification and "aberrant" E-cadherin staining patterns in invasive mammary carcinomas.

Case	In-situ	E-cadherin	Invasive	E-cadherin	Metastasis	E-cadherin
1	Lobular	Negative	Ductal	Negative	Lobular	Negative
2	Ductal	Pos, ++	Ductal	Pos, +	Lobular	Pos, +++
	Lobular	Negative				
3	Lobular	Negative	Ductal	Pos, +	CK-pos	Not Done
			Ductal	Negative		
4	Lobular	Not Done	Lobular	Pos, +++*	Lobular	Pos, +++
5	Lobular	Negative	Lobular	Negative	None	

*in pleomorphic areas. Weak, discontinuous positivity was seen in more classical areas of invasive carcinoma in the same tumor.

Pos-positive; + weak, ++ moderate, and +++ strong cell membrane immunoreactivity.

### Case 1

A 62 year-old woman underwent a left lumpectomy in April of 2001 which revealed invasive carcinoma, histologically classified as ductal type.

#### Microscopic findings

**Figure 1:**
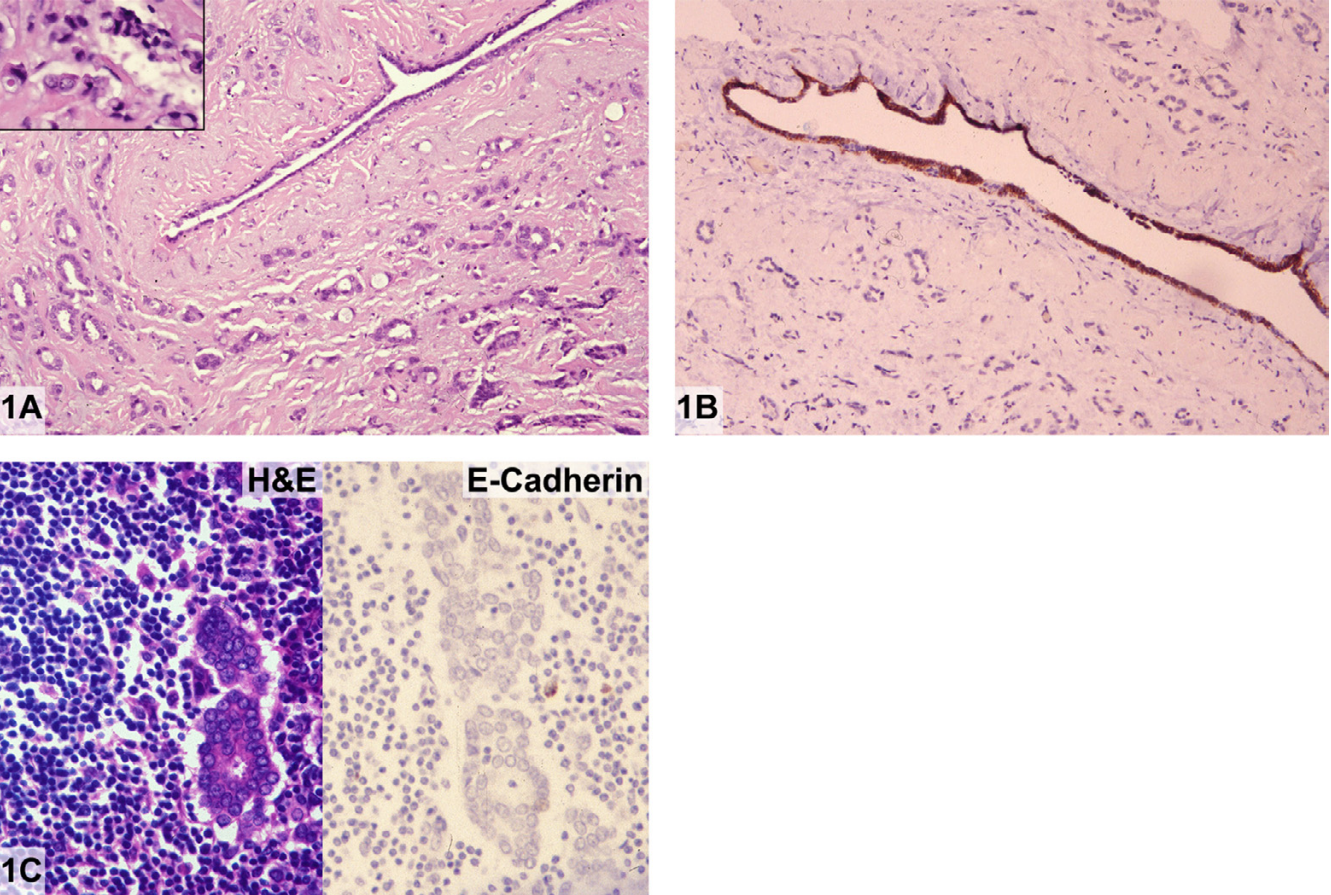
Absence of E-cadherin in invasive "ductal" carcinoma (case 1). **A: **Moderately differentiated invasive ductal carcinoma. Focally, tumor cells are seen growing in a linear fashion and contain intracytoplasmic mucin vacuoles (inset) (Hematoxylin and eosin). **B: **Invasive ductal carcinoma exhibiting complete absence of reactivity for E-cadherin. Normal duct serves as an internal positive control (Immunoperoxidase stain for E-cadherin). **C: **Metastatic carcinoma with glandular differentiation (Hematoxylin and eosin, left) showing complete absence of E-cadherin immunoreactivity (Immunoperoxidase stain for E-cadherin, right).

The 2.3 cm invasive carcinoma was well to moderately differentiated with intermediate nuclear grade. Well-formed ducts comprised greater than 90% of the invasive carcinoma (Figure 1A). In some areas, tumor cells assumed a linear growth pattern and contained intracytoplasmic mucin vacuoles (Figure 1A, inset). The invasive carcinoma was estrogen and progesterone receptor positive by immunohistochemistry. Lobular carcinoma in-situ (LCIS), mostly of the classical type with some foci having pleomorphic features and apocrine cytology was also present. In areas, classical LCIS extended into adjacent ducts in a pagetoid distribution. A single axillary lymph node was involved by metastatic carcinoma composed of tumor cells in a diffuse, discohesive pattern characteristic of metastatic lobular carcinoma. Focal gland formation was also evident in this metastatic deposit. Intracytoplasmic mucin vacuoles were readily identified within tumor cells.

An E-cadherin immunohistochemical stain was performed on representative samples of invasive, in-situ, and metastatic carcinoma. No cell membrane reactivity was demonstrated in the primary invasive carcinoma or in the nodal metastasis including areas of glandular differentiation (Figure 1B and 1C). Complete absence of E-cadherin immunoreactivity was also seen in foci of LCIS.

In this case, aberrant E-cadherin protein expression was represented by absence of cell membrane immunoreactivity in gland-forming regions of invasive as well as metastatic carcinoma which appeared to be phenotypically "ductal".

### Case 2

A 60 year-old female presented with a left breast mass. Subsequent excisional biopsy revealed invasive duct carcinoma.

#### Microscopic findings

**Figure 2:**
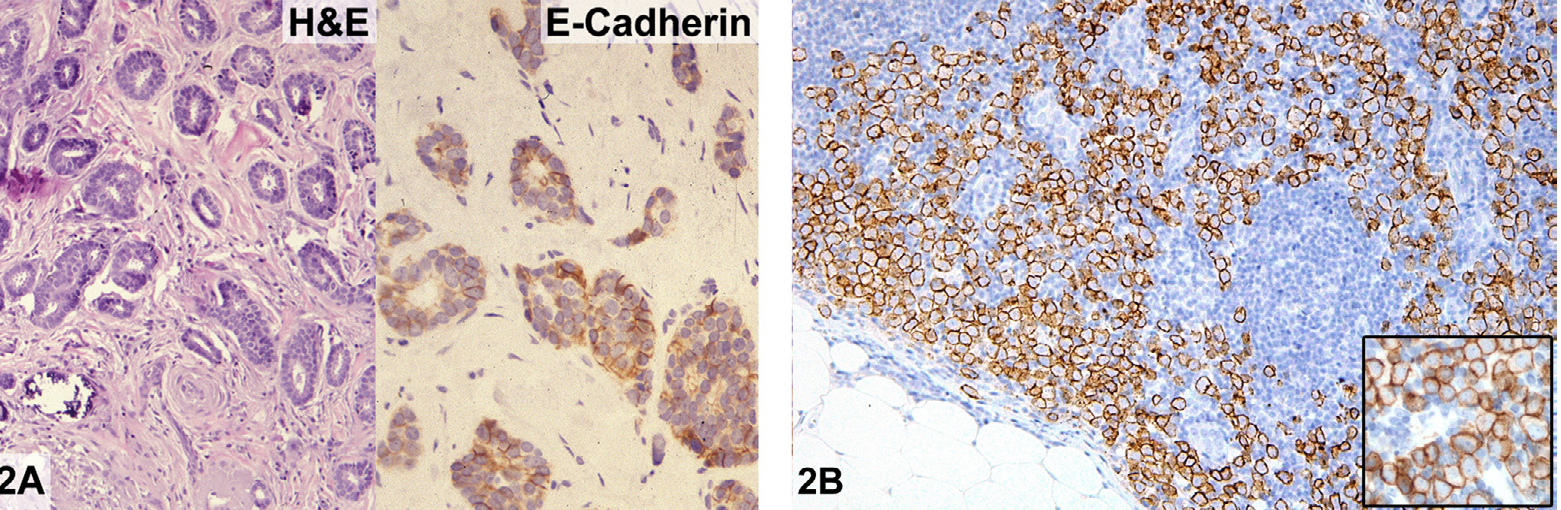
E-cadherin reactivity in metastatic "lobular" carcinoma (case 2). **A: **Well-differentiated invasive ductal carcinoma (Hematoxylin and eosin, left) exhibiting weak cell membrane positivity for E-cadherin (Immunoperoxidase stain for E-cadherin, right). **B: **Nodal metastasis demonstrating strong cell membrane immunoreactivity for E-cadherin; higher magnification of tumor cells (inset) (Immunoperoxidase stain for E-cadherin).

The lumpectomy specimen contained an 8 mm invasive well-differentiated duct carcinoma. Although greater than 90% of the tumor exhibited glandular differentiation, very focal areas with a single cell and linear growth pattern were also present (Figure 2A). The invasive carcinoma was estrogen and progesterone receptor positive and negative for HER-2/neu protein overexpression by immunohistochemistry. In-situ carcinoma was ductal in type with cribriform architecture and low nuclear grade arising predominantly in a background of typical and atypical columnar cell hyperplasia. Classical LCIS that expanded lobules with central discohesion was also present. The re-excisional specimen revealed additional foci of LCIS and no invasive carcinoma. An ipsilateral sentinel lymph node biopsy was performed. The lymph node was involved by metastatic carcinoma with a diffuse, discohesive growth pattern characteristic of lobular carcinoma. A minority of the tumor (<5%) also showed glandular differentiation.

The immunohistochemical stain for E-cadherin showed weak cell membrane reactivity in areas of the primary invasive carcinoma exhibiting a ductal phenotype (Figure 2A). In contrast, areas of invasive carcinoma with a linear, "lobular" phenotype displayed strong, uniform cell membrane staining (not shown). Diffuse, strong and uniform immunoreactivity was also seen in the nodal metastasis (Figure 2B).

Foci of intraductal carcinoma demonstrated moderate positivity for E-cadherin while those of classical LCIS were negative for this antigen.

In this case, aberrant E-cadherin protein expression consisted of weak immunoreactivity in the "ductal" areas of invasive carcinoma, whereas strong positivity was appreciated in areas of invasive and metastatic carcinoma with a lobular histologic phenotype.

### Case 3

A 62 year-old woman presented with a right breast mass. Stereotactic needle core biopsy revealed an invasive, moderately differentiated carcinoma that was classified as ductal. Subsequent wide local excision included the needle core biopsy site as well as an area of radiographic density not previously sampled.

#### Microscopic findings

The excisional biopsy revealed two separate invasive tumors measuring 8 and 5 mm, respectively. Well-formed glands comprised 80–90% of both tumors. Adjacent classical LCIS was also present. The larger tumor was strongly positive for estrogen and progesterone receptors by immunohistochemistry. No membrane expression for HER-2/neu was appreciated. A single ipsilateral sentinel lymph node contained several cytokeratin-positive cells. Five additional axillary lymph nodes were free of metastatic disease.

**Figure 3:**
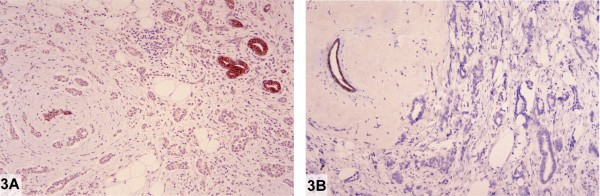
Weak and absent E-cadherin reactivity in two concurrent invasive "ductal" carcinomas (case 3)**. A: **Larger invasive ductal carcinoma exhibiting weak staining for E-cadherin. Normal duct serves as an internal positive control (Immunoperoxidase stain for E-cadherin). **B: **Smaller invasive ductal carcinoma showing complete absence of staining for E-cadherin. Normal duct serves as an internal positive control (Immunoperoxidase stain for E-cadherin).

The immunohistochemical stain for E-cadherin of the larger tumor was weak and discontinuous (Figure 3A). There was no E-cadherin cell membrane reactivity in the smaller invasive carcinoma (Figure 3B) or in adjacent LCIS (not shown). An E-cadherin stain was not performed on the sentinel lymph node.

Aberrant E-cadherin protein expression in this case consisted of predominantly negative immunoreactivity in invasive tumors exhibiting a ductal histological phenotype.

#### Case 4

A 57 year-old woman presented with a mass in the left breast.

#### Microscopic findings

**Figure 4:**
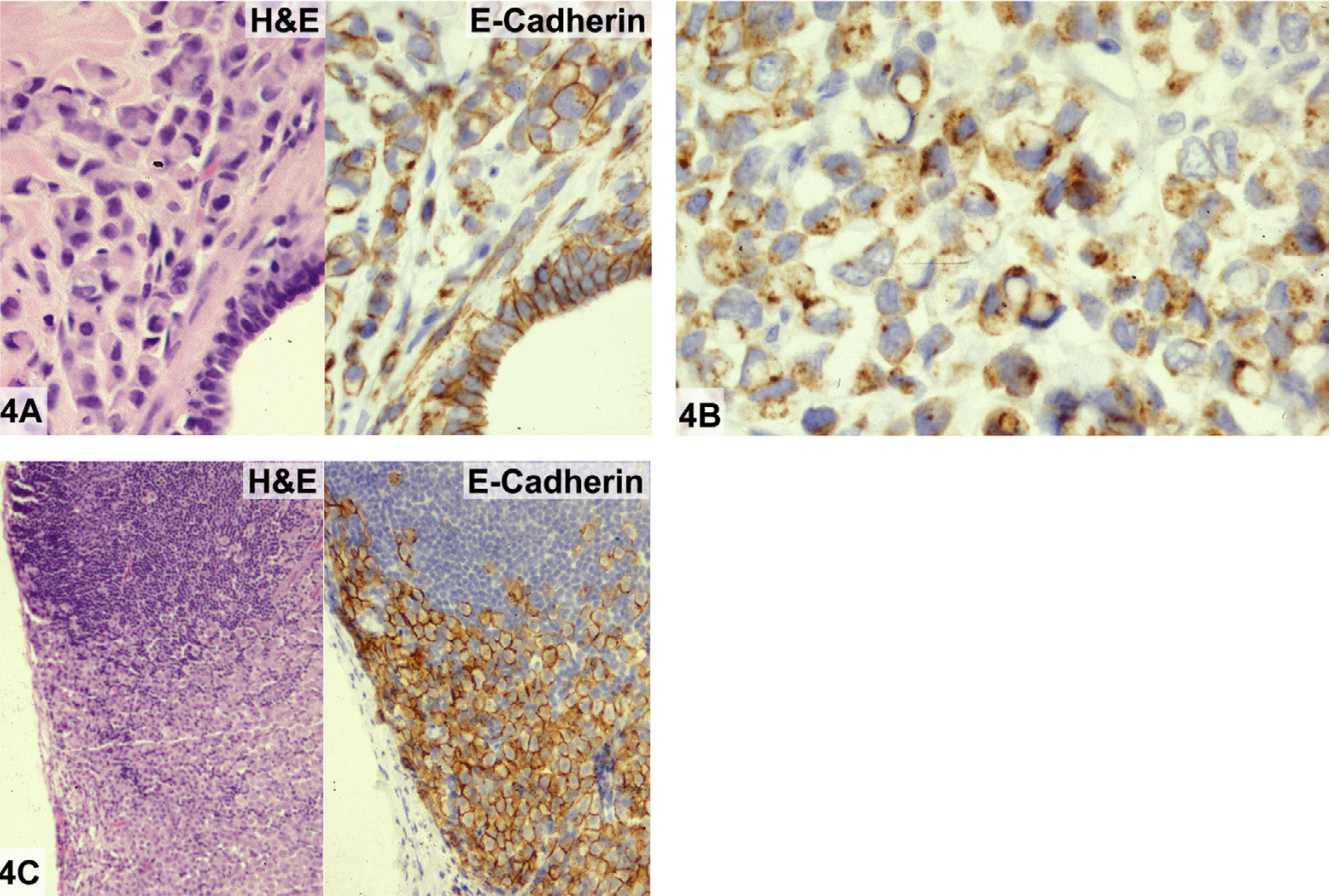
E-cadherin reactivity in invasive "lobular" carcinoma (case 4). **A: **Invasive carcinoma with pleomorphic lobular features showing predominantly alveolar and solid growth patterns and intermediate-high nuclear grade (Hematoxylin and eosin, left). Strong cell membrane E-cadherin reactivity in tumor cells and in normal adjacent ductal epithelium (Immunoperoxidase for E-cadherin, right). **B: **Some tumor cells demonstrate a punctate staining pattern within the cell membrane or in the cytoplasm (Immunoperoxidase for E-cadherin). **C: **Metastatic carcinoma with lobular characteristics involving an ipsilateral axillary lymph node (Hematoxylin and eosin, left). Tumor cells showing strong cell membrane reactivity for E-cadherin (Immunoperoxidase for E-cadherin, right).

The excised specimen contained a 2.9 cm invasive and in-situ poorly differentiated carcinoma. More than 90% of the tumor consisted of invasive carcinoma which exhibited predominantly alveolar and solid growth patterns, absence of gland formation, and intermediate-high nuclear grade (Figure 4A, left). *In-situ *carcinoma comprised the remainder of the tumor mass and had a solid growth pattern, apocrine features, and intermediate-high nuclear grade. Both invasive and in-situ carcinoma formed signet-ring cells containing bluish-tinged, intracytoplasmic mucin vacuoles and together with the nuclear atypia, had the appearance of pleomorphic lobular carcinoma. The invasive carcinoma showed nuclear reactivity for estrogen and progesterone receptors by immunohistochemistry. The subsequent left mastectomy specimen revealed residual invasive carcinoma. A single, 2.2 cm left axillary lymph node contained metastatic carcinoma with extranodal extension. Morphologically, the metastatic deposit showed a "lobular phenotype" in the form of signet-ring cells growing in a diffuse distribution and absence of glandular differentiation (Figure 4C, left).

An E-cadherin immunohistochemical stain performed on the primary invasive carcinoma revealed a heterogeneous staining pattern. Tumor areas with alveolar and solid growth patterns exhibited strong, continuous cell membrane immunoreactivity (Figure 4A, right) while those having a linear growth pattern had relatively weaker, discontinuous positivity (not shown). Additionally, some tumor cells demonstrated a punctate staining pattern either within the cell membrane or cytoplasm (Figure 4B). Overall, the E-cadherin reactivity was interpreted as positive in areas of invasive carcinoma despite heterogeneous staining in some areas. Metastatic carcinoma involving the lymph node demonstrated strong, continuous membrane staining (Figure 4C, right).

Aberrant staining in this case consisted of strong E-cadherin protein expression in areas of primary invasive carcinoma morphologically resembling pleomorphic lobular carcinoma. Weaker, discontinuous E-cadherin positivity was observed in other areas of the invasive carcinoma resembling classical type of lobular carcinoma. Punctate membrane or cytoplasmic staining by E-cadherin was also observed in some tumor cells. The lymph node metastasis which also had a "lobular" appearance had strong membrane E-cadherin reactivity similar to that seen in the primary invasive carcinoma.

### Case- 5

A 37 year-old woman presented with a left breast mass. Stereotactic needle core biopsy revealed in-situ and invasive, moderately differentiated carcinoma that was classified as ductal. Subsequent wide local excision of the mass was performed.

#### Microscopic findings

The excisional biopsy revealed an invasive tumor measuring 1.9 cm. The invasive carcinoma predominantly exhibited a classical lobular pattern with well-formed glands comprising 20% of the tumor. Extensive multifocal classical LCIS, focally florid type was also present. The tumor was positive for estrogen and progesterone receptors by immunohistochemistry. No membrane expression for HER-2/neu was present.

**Figure 5:**
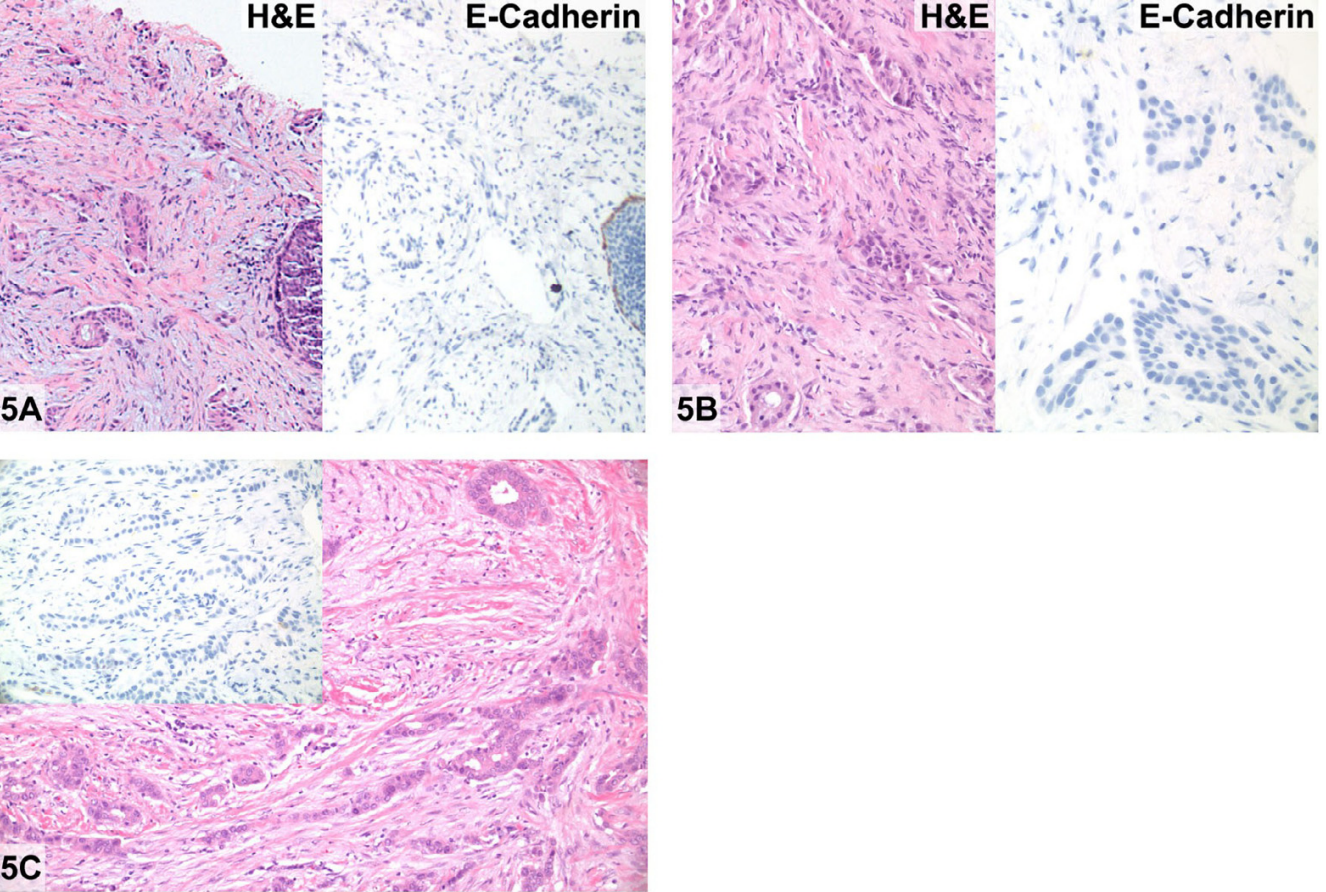
Invasive lobular carcinoma with glandular "ductal" differentiation (case 5). **A: **Moderately differentiated invasive "ductal" carcinoma and carcinoma in situ on core biopsy (Hematoxylin and eosin, right) showing complete absence of E-cadherin immunoreactivity. (Immunoperoxidase stain for E-cadherin, left). **B: **No immunoreactivity for E-cadherin (Immunoperoxidase stain for E-cadherin, left) confirms invasive lobular carcinoma with glandular differentiation in the excision biopsy (Hematoxylin and eosin, right). **C**: Invasive lobular carcinoma with gland formation (Hematoxylin and eosin, right and immunoperoxidase stain for E-cadherin, left).

There was no E-cadherin cell membrane reactivity in the invasive component, both phenotypically lobular and ductal areas (Figure 5) as well as in adjacent LCIS (Figure 5A).

Aberrant E-cadherin protein expression in this case consisted of complete absence of immunoreactivity in an invasive tumor with areas of unquestionable glandular differentiation.

## Discussion

E-cadherin is a transmembrane glycoprotein that mediates calcium- dependent cell-cell adhesion and is expressed mainly in epithelial cells and thought to play a critical role in epithelial differentiation and morphogenesis [10,11]. Mutations in the E-cadherin gene (CDHI) located on chromosome 16q22.1 have been demonstrated in gastric, ovarian, endometrial and thyroid carcinomas in addition to lobular breast carcinomas [12]. More recently molecular alteration in the E-cadherin gene resulting in loss of expression of E-cadherin in in situ and invasive lobular carcinomas has been demonstrated by molecular studies [12-16]. Somatic mutations in CDH1 occur in lobular breast carcinomas with a frequency ranging from 10–56% (15–20% of invasive lobular carcinomas) and are rare in ductal carcinomas [17,18]. Molecular alteration with loss of heterozygosity at 16q21.1 is the most frequent chromosome alteration in lobular carcinoma and correlates with the loss of E-cadherin expression [19]. In lobular carcinomas and diffuse type of gastric carcinomas absence of E-cadherin expression has been suggested to contribute to discohesive, single cell and infiltrative growth characteristic of these tumors [20,21].

Several studies have demonstrated the reliability of E-cadherin as a marker for distinguishing ductal from lobular carcinoma. In a study by Acs *et al*, where 183 invasive (duct, lobular and mixed) and 198 in situ carcinomas were studied, all *in situ *and invasive ductal carcinomas showed strong membrane E-cadherin expression. Forty-one of 42 invasive and 50 of 53 in situ lobular carcinoma showed complete absence of E-cadherin expression [1]. Moll *et al*, studied 89 primary infiltrating carcinomas immunohistochemically using an antibody to E-cadherin and found that 78% of well and moderately differentiated invasive duct carcinomas (IDC) showed strong linear staining for E-cadherin. Fifty-four percent of the poorly differentiated IDC however, had reduced and heterogeneous staining. E-cadherin reactivity was absent in 86.4% of the invasive lobular carcinomas and there was weak staining in 13.6 %. Among the in situ carcinomas the majority of DCIS had strong E-cadherin expression and all cases of LCIS showed absence of staining [7].

We have separately studied a series of 132 breast carcinomas, both in situ and invasive, for E-cadherin expression [22]. In this series there was 100% correlation between classification as ductal or lobular on the basis of conventional histologic criteria and E-cadherin reactivity. In the course of this study we observed some examples of in situ lobular carcinoma involving ducts and lobules which contained residual non-neoplastic E-cadherin positive epithelial and myoepithelial cells. In some instances, especially when there was pagetoid spread of LCIS, staining of non-neoplastic cells could be mistaken for reactivity in the in situ carcinoma. We also have seen cases where in situ lobular carcinoma displayed fragmented, discontinuous and usually weak reactivity. These phenomena probably account for most of the reported instances of lobular carcinoma with E-cadherin reactivity.

Few studies have specifically investigated invasive carcinomas with mixed duct and lobular features (IDLC). Acs *et al*, investigated 41 cases which were classified as such due to the presence of a "mixture in variable degrees of ductal cytology and growth pattern (large, pleomorphic cells with cohesive cellular arrangement with or without lumen formation) and typical lobular pattern (dispersed infiltrating fashion) in the same lesion, or to occasional tubules or small nest formation in a lesion that otherwise showed a typical lobular type of infiltration and cytology" [1]. They correlated the E-cadherin immunohistochemical staining patterns with morphologic features (presence/absence of tumor cell nests and trabeculae, tubule/lumen formation, intracytoplasmic lumina, discohesion) in these cases and found three patterns of E-cadherin expression. Ten cases grouped as "lobular like" IDLC showed similar absence of staining as seen in traditional invasive lobular carcinomas while twenty-four "ductal like" IDLCs had uniform membrane positivity similar to that seen in typical ductal carcinomas. The accompanying in situ carcinoma was mainly LCIS and DCIS, respectively, in these groups. A third group comprising the remaining 7 cases deemed as the "intermediate" IDLC, demonstrated focal, complete loss of E-cadherin staining. These tumors were accompanied by LCIS and DCIS. Statistical analysis showed that complete loss of immunostaining correlated well with the histologic impression of lobular features and lack of tubule or lumen formation exhibited by IDLC. Acs et al concluded that all 10 "lobular like" and 24 "ductal like" examples of infiltrating carcinomas demonstrated E-cadherin expression similar to typical lobular and ductal carcinomas, respectively, and therefore, could be further classified based on their immunophenotype for E-cadherin. However, 7 (3.8%) of 182 invasive carcinomas in their study remained histologically and immuno phenotypically mixed ductal and lobular carcinoma [1]. These latter cases demonstrated real heterogeneity in E-cadherin expression, however, the authors noted that areas of tumor showing complete lack of membrane staining correlated well with the histologic impression of lobular features exhibited by the tumor.

Goldstein *et al*, investigated 80 mixed (lobular and ductal) breast carcinomas. They found that the percentage of strongly E-cadherin-reactive lobular carcinoma cells was greatest in the mixed, predominantly ductal carcinomas and the greatest numbers of strongly E-cadherin-stained lobular carcinoma cells were identified near the periphery of the mixed carcinomas, particularly along the leading edge of these tumors. In addition, they observed that the nuclear grade of the lobular carcinoma component increased as the percentage of the carcinoma that was of the ductal type increased [9]. In the small series that we studied [22], four carcinomas with duct and lobular features were E-cadherin positive. These divergent results indicate that further work needs to be done to determine how E-cadherin immunostaining can be used for the classification of structurally heterogeneous tumors.

The cases described in the present study represent a phenomenon wherein the histological structural phenotype did not match the "genotype" as suggested by the staining pattern of the E-cadherin immunohistochemical stain. This "aberrant" staining pattern is exceedingly rare in our experience. In cases 1, 2, 3, and 5 invasive carcinomas with predominantly gland forming elements and therefore ductal phenotype lacked E-cadherin reactivity. The differential diagnostic option in cases 1–3 and 5 is tubulolobular carcinoma based on the hybrid morphology of gland (tubules) and linear (lobular) growth pattern. Tubulolobular carcinoma is a distinct subtype of mammary carcinoma, which were originally thought to represent a tubular variant of lobular carcinoma. Few studies have examined the immunoprofile of tubulolobular carcinoma with respect to the E-cadherin staining pattern. In a recent study by Wheeler et al in which 27 cases of tubulolobular carcinoma composed of intermixed, round to angulated tubules and single file cell cords with diffuse and targetoid growth pattern demonstrated strong and diffuse positivity for E-cadherin in both the cell cord and tubular components [23]. In our experience, mammary carcinomas with duct and lobular features also showed E-cadherin positivity. The diffuse E-cadherin positive staining in tubulolobular mammary carcinoma appears to support a ductal differentiation rather than lobular origin However, in cases 1–3 and 5 the E-cadherin immunostaining in the glandular components was negative or weakly positive. In the fourth case, the poorly differentiated invasive carcinoma with pleomorphic lobular features exhibited an unusual and heterogeneous pattern of E-cadherin expression. In addition to strong cell membrane positivity, there was also cytoplasmic as well as membrane dot-like E-cadherin staining of tumor cells.

Cytoplasmic E-cadherin reactivity has been described in diffuse type of gastric adenocarcinomas as well as in some invasive lobular carcinomas [13,21,24]. Acs and co-workers encountered a single case of E-cadherin-positive invasive lobular carcinoma which was histologically compatible with pleomorphic lobular carcinoma and associated with intermediate grade solid DCIS. The authors surmised that this tumor was most likely an example of ductal carcinoma with a dispersed growth pattern. In addition, peri-nuclear dot staining by E-cadherin was seen in two cases of LCIS. Such aberrant staining may be due to mutant E-cadherin, which is incorrectly processed within the Golgi apparatus, or from accelerated protein turnover [1]. Kowalski *et al*., reported that all eight invasive lobular carcinomas showed only cytoplasmic staining for E-cadherin which was also localized to the cytoplasm in the majority of (7/9) metastatic lobular carcinomas [21]. In gastric examples, it has been suggested that cytoplasmic localization was due to abnormal transport mechanisms which were also present in the non-malignant gastric epithelium. This observation was taken to be evidence that alteration in E-cadherin occurs early in the development of gastric carcinogenesis. This finding was not observed in the non-neoplastic breast tissue [24].

Molecular studies have shown that invasive breast cancer is a disease with multiple cytogenetic subclones which are also present in the preinvasive lesions [25]. The two major types of breast cancer exhibit genetic heterogeneity, but occasionally a small percentage of DCIS cases are accompanied by invasive lobular carcinoma and similarly, LCIS cases may develop invasive carcinoma of the ductal or lobular type [26]. Transition from LCIS towards ductal invasive carcinoma or from DCIS towards lobular invasive carcinoma is possible [25]. Molecular analyses of pure tubular carcinomas using comparative genomic hybridization suggest that lobular and tubular carcinomas share an early genomic change, with a similar mechanism of progression [27]. The aberrant staining patterns reported in this study may be due to non-functional E-cadherin protein. Disruption of E-cadherin signaling with inactivation of E-cadherin protein by phosphorylation by activation of SRC family kinases and several growth factor receptors has been previously reported. [28]. Normal E-cadherin expression can be seen despite compromised functional ability via defects in catenin (alpha, beta and gamma) [29] Loss of E-cadherin expression can occur by gene deletion, as well as defects in transcription and methylation [30]. E-cadherin gene transcription is inhibited by two zinc-finger transcription factors SLUG and SNAIL, which may cause transient down-regulation and up-regulation of E-cadherin in primary and metastatic breast carcinoma [31].

We speculate that E-cadherin may play a critical role in molecular classification of breast carcinoma and may further elucidate the origin of tumors with a mixed or discordant phenotype.

## Conclusion

Findings such as those illustrated in this study occur in virtually all biologic phenomena and they do not invalidate the very high degree of correlation between the expression of E-cadherin and the classification of breast carcinomas as ductal or lobular type on the basis of conventional histologic criteria. Further studies are necessary to clarify whether E-cadherin protein is non-functional or truly represents exceptional biology in breast carcinomas exhibiting aberrant staining of E-cadherin.

## Abbreviations

IDLC – invasive carcinomas with mixed duct and lobular features

LCIS – lobular carcinoma in situ

DCIS – ductal carcinoma in situ

IDC – infiltrating ductal carcinoma

## Competing interests

The author(s) declare that they have no competing interests.

## Authors' contributions

**MH, SS, MM**, and **PPR**: These authors contributed equally to the preparation of this manuscript.

**ST **and **EB**: Each of these authors contributed one case.
